# Etiologic spectrum and occurrence of coinfections in children hospitalized with community-acquired pneumonia

**DOI:** 10.1186/s12879-017-2891-x

**Published:** 2017-12-20

**Authors:** Wujun Jiang, Min Wu, Jing Zhou, Yuqing Wang, Chuangli Hao, Wei Ji, Xinxing Zhang, Wenjing Gu, Xuejun Shao

**Affiliations:** 1 Department of Respiratory Medicine, Children’s Hospital of Soochow University, Suzhou, China; 2 Department of Clinical Laboratory, Children’s Hospital of Soochow University, Suzhou, China

**Keywords:** Community-acquired pneumonia, Children, Coinfection

## Abstract

**Background:**

Co-infections are common in childhood community acquired pneumonia (CAP). However, their etiological pattern and clinical impact remains inconclusive.

**Methods:**

Eight hundred forty-six consecutive children with CAP were evaluated prospectively for the presence of viral and bacterial pathogens. Nasopharyngeal aspirates were examined by direct immunofluorescence assay or polymerase chain reaction (PCR) for viruses. PCR of nasopharyngeal aspirates and enzyme-linked immunosorbent assays were performed to detect M. pneumoniae. Bacteria was detected in blood, bronchoalveolar lavage specimen, or pleural fluid by culture.

**Results:**

Causative pathogen was identified in 70.1% (593 of 846) of the patients. The most commonly detected pathogens were respiratory syncytial virus (RSV) (22.9%), human rhinovirus (HRV) (22.1%), M. pneumoniae (15.8%). Coinfection was identified in 34.6% (293 of 846) of the patients. The majority of these (209 [71.3%] of 293) were mixed viral-bacterial infections. Age < 6 months (odds ratio: 2.1; 95% confidence interval: 1.2–3.3) and admission of PICU (odds ratio: 12.5; 95% confidence interval: 1.6–97.4) were associated with mix infection. Patients with mix infection had a higher rate of PICU admission.

**Conclusions:**

The high mix infection burden in childhood CAP underscores a need for the enhancement of sensitive, inexpensive, and rapid diagnostics to accurately identify pneumonia pathogens.

## Background

Childhood community-acquired pneumonia (CAP) is the leading cause of mortality in children aged less than 5 years. Pneumonia causes almost 1 in 5 under-five deaths worldwide: more than 2 million children each year [1–3]. Despite this large disease burden, critical gaps in our knowledge about pediatric pneumonia remain [4], especially establishing the cause of pneumonia, because distinguishing possible prolonged shedding or colonization from active infection can be difficult.

In China, incidence of CAP ranged from 0.06-0.27 episodes per person-year and mortality ranged from 184 to 1223 deaths per 100,000 population for children <5 years [5]. The most common pathogens of CAP are viruses, followed by bacteria and atypical bacteria [6]. Co-infections are common in childhood CAP, especially in younger children [6, 7]. Previous studies from western countries revealed that the coinfection rate ranged from 23% to 35% [6, 8–11]. These studies showed appreciable differences in the frequencies of causative agents, which seemed to be related to seasonal, geographical, and racial factors. A study conducted in eight eastern cities in China revealed the mix infection rate was 14.4% [12]. However, in their study, they included a combination of traditional Chinese medicine and western medicine hospitals. Mix infection rate was significantly lower in traditional Chinese medicine hospitals compared with western medicine hospitals (1% vs 28.5%). As a matter of fact, there has been few well-defined prospective studies on the etiology and occurrence of coinfections of childhood CAP in China.

The objective of this study was to investigate the etiologic spectrum and occurrence of coinfections in children with CAP. Furthermore, the study provides data that will facilitate age-appropriate antibiotic selection and candidate vaccines for CAP.

## Methods

### Subjects

Eight hundred forty-six consecutive children with CAP admitted to Children’s Hospital of Soochow University were evaluated prospectively from January 2015 through Dec 2015. Children’s Hospital of Soochow University is a 1000 bed tertiary referral teaching hospital located in southeastern Jiangsu Province of East China. It has over 50,000 patients admitted to hospital each year. Children were included in this study if they were 1 month to 14 years old, had preceding fever (defined as body temperature ≥ 38 °C), and had clinical (chest retractions, tachypnea, nasal flaring, hypoxia, or abnormal auscultatory findings) and radiologic evidence of CAP. Children were excluded if they had preterm birth ≤34 weeks’ gestation, recent hospitalization (4 weeks before admission), immunodeficiency, history of a diagnosis of chronic lung disease, or congenital heart disease.

This study was approved by the Medical Ethics Committee of Children’s Hospital of Soochow University. The parents of the children enrolled in this study gave written informed consent before enrollment.

### Specimen collection

Nasopharyngeal aspirates were obtained from all the patients enrolled within 24 h after admission. A suction catheter was used to passed through the nose into the lower part of the pharynx. The depth of penetration was set at 7-9 cm. A total of 2 ml nasopharyngeal aspirates was obtained and sent for analysis within 30 min. It is centrifuged at 500×g for 10 min and resuspended in 2 ml saline and divided into 2 aliquots for pathogen detection using direct immunofluorescence assay (DFA) and PCR.

Blood, Pleural fluid (if indicated), and bronchoalveolar (BAL) (if indicated) specimens obtained for clinical care were also collected.

### Viral detection

DFA was used to detect syncytial virus infection (RSV), influenza virus A (IVA), influenza virus B (IVB), parainfluenza virus (PIV) I, PIV II, PIV III, and adenovirus (ADV). All assay kits were purchased from Chemicon (USA) and all staining procedures were performed according to the manufacturer’s instructions. Immunostained preparations were viewed with a fluorescence microscope (Leica 020-518.500, Germany).

RNA was extracted from nasopharyngeal specimens using Trizol (Invitrogen, USA). cDNA was synthesized by reverse transcription. The cyclic temperature settings were 94 °C, 30 s; 55 °C, 30 s; 68 °C, 30 s; amplified by 45 cycles with the last at 68 °C for 7 min. Human metapneumovirus (hMPV) and rhinoviruses (HRV) was assayed by fluorescent real-time PCR (BIO-RAD iCycler). For hMPV detection, primers were designed to specifically amplify the N gene (213 bps). The forward and reverse primers were hMPV-F: 5’-AACCGTGTACTAAGTGATGCACTC-3′ and hMPV-R: 5’-CATTGTTTGACCGGCCCCATAA-3′, respectively. For HRV detection, the primers and probe sequences were HRV-F: 5’-TGGACAGGGTGTGAAGAGC -3′; HRV-R:5’-CAAAGTAGTCGGTCCCATCC-3′; HRV-probe: FAM-TCCTCCGGCCCCTGA ATG-TAMRA. The cyclic temperature settings were 94 °C, 30 s; 56 °C, 30 s; 72 °C, 30 s; amplified, 40 cycles.

Nasopharyngeal specimen DNA was extracted as described above, and human bocavirus (hBoV)-DNA was detected by real-time fluorescent PCR. For HBoV VP1 gene detection, the primers and probe sequences were HBoV-F: 5’-TGACATTCAACTACCAACAACCTG-3’;HBoV-R:5’-CAGATCCTTTTCCTCCTCCAATAC-3′;HBoV-probe: FAM-AGCACCACAAAACACCTCAGGGG-TAMRA. The cyclic temperature settings were 94 °C, 30 s; 56 °C, 30 s; 72 °C, 30 s; amplified by 40 cycles.

### Bacterial detection

A bacterial pathogen was defined as detection of H. influenza, M.catarrhalis and other Gram-negative bacteria, *S. aureus*, S. anginosus/mitis, S. pneumoniae, or S. pyogenes in blood, BAL specimen, or pleural fluid by culture, or a significant rise in M. pneumoniae IgG, or the presence of IgM antibodies together with M. pneumoniae DNA. Other bacteria were considered contaminants according to the previous study [6].

A ELISA kit (Serion ELISA classic M. pneumoniae IgG/IgM, Würzburg, Germany) was used to detect M. pneumoniae IgM and IgG antibodies in paired serum samples of patients on admission and one week after admission, respectively. The cut-off value of the test was 0.5 × mean optical density (OD) of the kit control serum. A significant rise in M. pneumoniae IgG titre was defined as a doubling of the OD value above the cut-off.

A quantitative diagnostic kit (DaAn Gene Co., Ltd. Guangzhou, China) for M. pneumoniae DNA was performed to identify the 16S rRNA gene of MP extracted from nasopharyngeal specimens. The primers and probe sequences were MP-F: 5’-GCAAGGGTTCGTTATTTG-3′ and MP-R: 5’-CGCCTGCGCTTGCTTTAC-3′, and MP-probe: FAM-AGGTAATGGCTAGAGTTTGACTG-TAMRA.

### Radiographic confirmation

A senior radiologist (G.W.L.), blinded to demographic and clinical information, reviewed all chest radiographs. The radiologist assigned standardized and mutually exclusive diagnoses that included unequivocal focal or segmental consolidation with or without pleural effusion, atelectasis, consolidation indistinguishable from atelectasis, or interstitial pneumonia.

### Statistical analysis

Statistical analyses were performed using the Statistical Package for the Social Sciences (version 17.0). Data were expressed as number with percentage, mean and standard deviation (SD) or, median and interquartile range as appropriate. Normally distributed continuous variables were compared using the Student t test and non-normally distributed variables were analyzed using Mann-Whitney U test. Categorical data were analyzed using the chi-squared (χ2) test or Fisher’s exact test. Posthoc multiple comparisons were performed to determine the origins of significant differences, and the results were adjusted by using the Bonferroni method. Backward stepwise logistic-regression analyses were performed to determine the best predictors of mix infections (viral and bacterial, viral and viral, bacterial and bacterial infections). Covariates included in the regression model were age (<6 month /≥6 months), gender, fever, wheeze, dyspnea, pleural effusion, WBC count, percentage of neutrophils, CRP, need of oxygen and pediatric intensive care unit (PICU) admission. Only variables from the univariate analyses that were significant at the .05 level were entered into the multivariate logistic-regression analyses. Goodness of fit of the regression model was tested with the Hosmer–Lemeshow test, with *P* > 0.05 considered to indicate lack of deviation between the model and observed event rate. The area under ROC curves (AUROC) of predictors for predicting mix infections were calculated. *P* value < .05 was considered statistically significant.

## Results

### Patients

Eight hundred forty-six children with CAP met the inclusion criteria for enrollment. Of these patients, 489 (57.8%) were male. Ages ranged from 1 month to 14 years (median: 11 months). Two hundred ninety-four (34.8%) children were <6 months old, 161(19%) were 6 months to <1 years old, 208 (24.6%) were 1 to <3 years old, 117 (13.8%) were 3 to <5 years old, and 66(7.8%) were ≥5 years old.

### Etiology

**Fig. 1:**
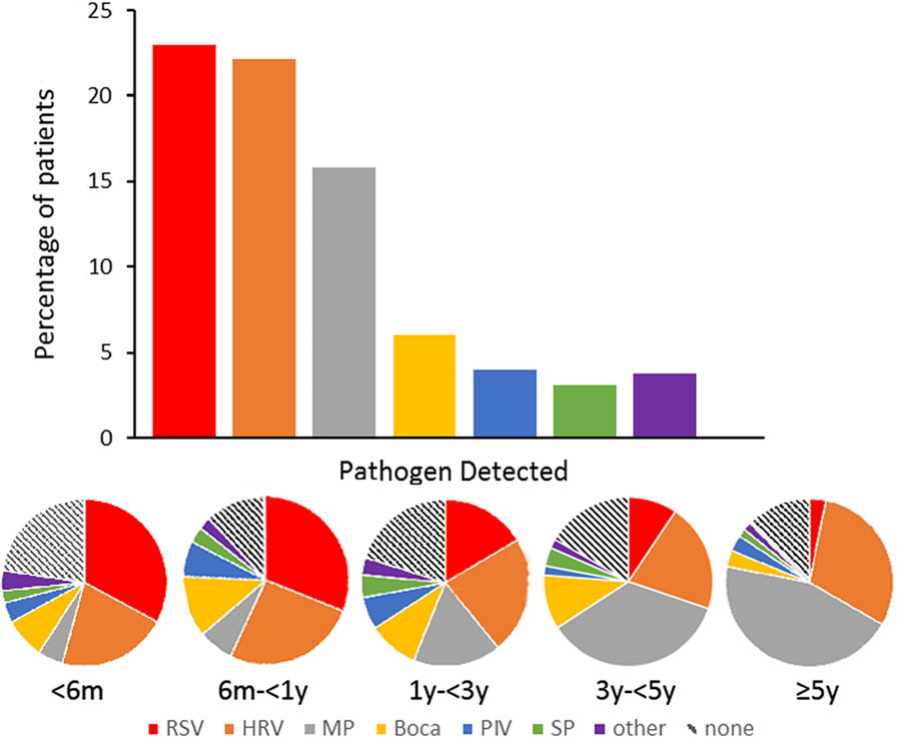
Pathogen detection among children with community acquired pneumonia requiring hospitalization. RSV indicates respiratory syncytial virus, HRV indicates human rhinovirus, MP indicates *M. pneumoniae*, Boca indicates bocavirus, PIV indicates parainfluenza 1–3, SP indicates *S. pneumoniae*

**Fig. 2:**
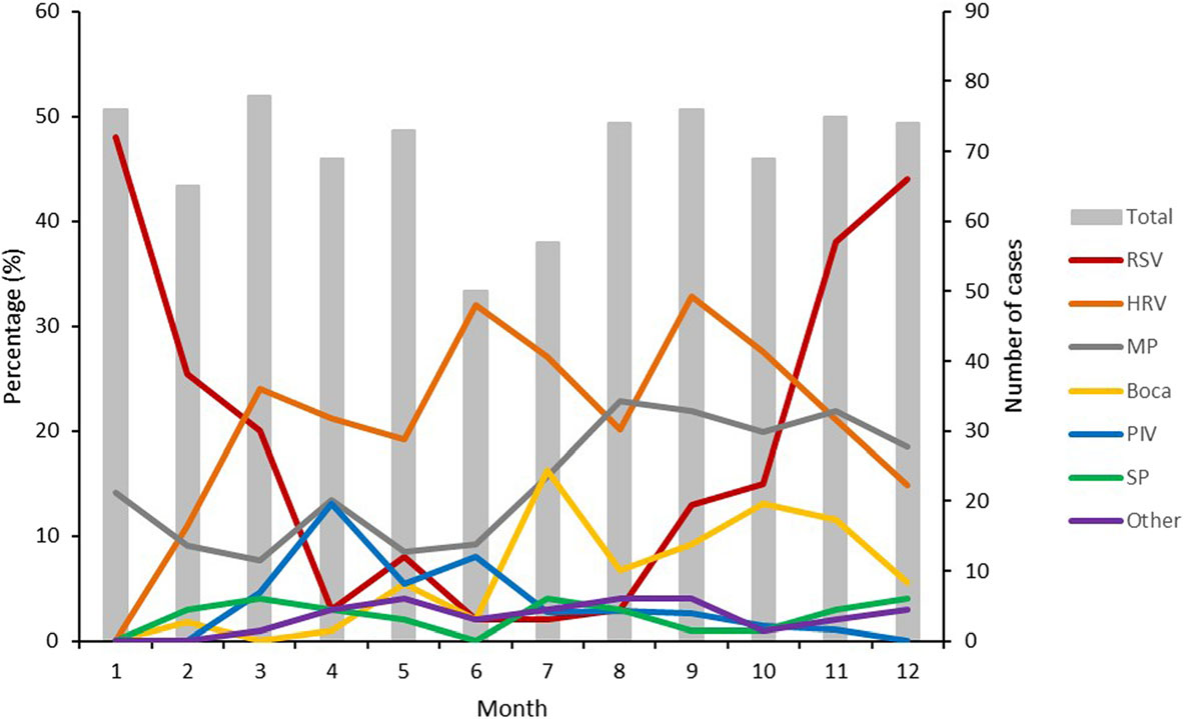
Monthly distributions of commonly detected pathogens were shown in Fig. 2. RSV indicates respiratory syncytial virus, HRV indicates human rhinovirus, MP indicates *M. pneumoniae*, Boca indicates bocavirus, PIV indicates parainfluenza 1–3, SP indicates *S. pneumoniae*

At least 1 respiratory pathogen was identified in 70.1% (593 of 846) of the patients. Pathogens were documented in 70.7% of patients <6 months old, 76.1% of patients 6 months to <1 years old, 70.2% of patients 1 to <3 years old, 74.0% of patients 3 to <5 years old, and 78.0% of patients ≥5 years old. The most commonly detected pathogens were RSV (22.9%), HRV (22.1%), M. pneumoniae (15.8%), hBoV (6.0%), PIV (4.0%) and S. pneumoniae (3.0%) (Fig. 1). RSV (24.6% vs. 3%, *P* < 0.01) was more commonly detected in children <5 years old compared with older children; M. pneumoniae (42.4% vs. 13.6%, *P* < 0.01) was more commonly detected in children ≥5 years old compared with younger children. Monthly distributions of commonly detected pathogens were shown in Fig. 2.

### Mixed infections

Coinfection was identified in 34.6% (293 of 846) of the patients. Of the 293 patients with coinfection, two pathogens were identified in 220 (75.1%) patients, three were identified in 60 (20.5%) patients and four in 13(4.4%) patients. The majority of these (209 [71.3%] of 293) were mixed viral-bacterial infections. Mixed viral-viral infections were identified in 56 (19.1%) patients and mixed bacterial -bacterial in 28 (9.6%) patients.

**Fig. 3:**
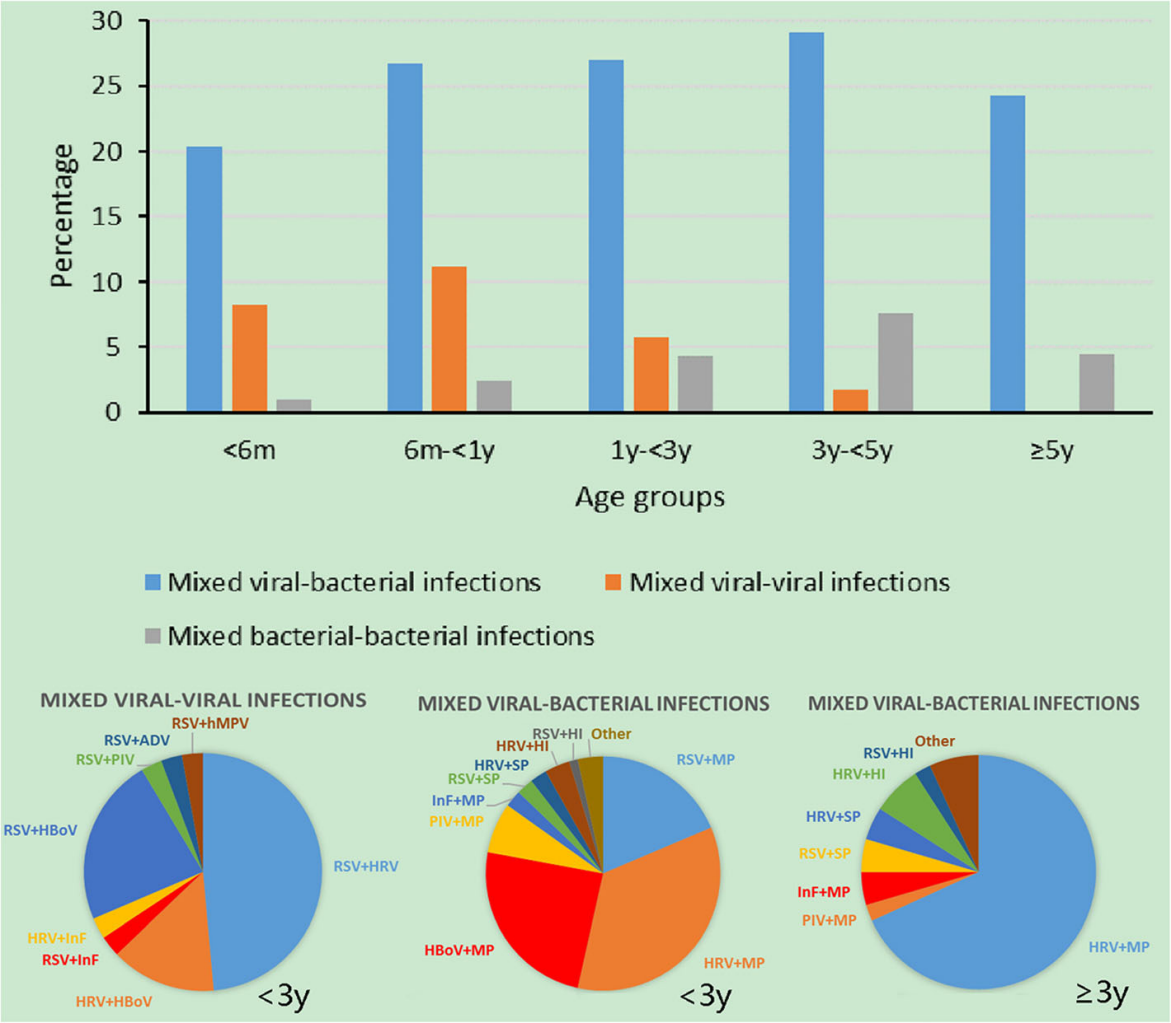
Distribution of pathogen coinfections associated with community acquired pneumonia, stratified by age. RSV indicates respiratory syncytial virus, HRV indicates human rhinovirus, MP indicates M. pneumoniae, HBoV indicates bocavirus, PIV indicates parainfluenza 1–3, SP indicates S, pneumoniae, ADV indicates adenovirus, InF indicates influenza virus A and B, hMPV indicates human metapneumovirus, HI indicates H. influenza

Coinfections were documented in 59.9% of patients <6 months old, 65.8% of patients 6 months to <1 years old, 60.6% of patients 1 to <3 years old, 52.1% of patients 3 to <5 years old, and 34.8% of patients ≥5 years old. Mixed viral-bacterial infections were the major coinfections in each age group. Mixed viral-viral infections were more commonly detected in children <3 years old compared with older children (9.2% vs 1.5%, *P* < 0.01) For children <3 years, RSV/HRV was the most common combination (48.6%) of viral-viral infections, followed by RSV/hBoV (22.9%) and HRV/hBoV (14.3%). HRV/ M. pneumoniae (34.9%) was the most common combination of viral-bacterial infections, followed by M. pneumoniae /hBoV (24.4%) and RSV/ M. pneumoniae (18.6%) (Fig. 3).

The coinfection status of varied pathogens is shown in Table 1. Co-infection was found significantly more frequently among patients with hBoV (64.7%), M. pneumoniae (51.5%) and HRV (47.6%) than those with RSV (30.9%) and PIV (23.5%) (*P* < 0.01, respectively).

**Table 1:** Pathogens Identified in Hospitalized Children with Community-Acquired Pneumonia

Pathogen	No. of Episodes			Total No. of Episodes
	Coinfection	Coinfection With Viruses^a^	Coinfection With Bacteria^a^	
Viruses^b^				
RSV	60 (30.9)	31 (16.0)	35 (18.0)	194
Rhinovirus	89 (47.6)	23 (12.3)	73 (39.0)	187
Bocavirus	33 (64.7)	13 (25.5)	21 (41.2)	51
Parainfluenza 1–3	8 (23.5)	1 (2.9)	8 (23.5)	34
Influenza A or B^c^	4	2	3	8
Metapneumovirus^c^	2	1	1	3
Adenovirus^c^	1	1	0	4
Bacteria^b^				
*M. pneumoniae*	69 (51.5)	63 (47.0)	14 (10.4)	134
*S. pneumoniae*	13 (52.0)	5 (20.0)	8 (32.0)	25
*H. influenzae*	7 (63.6)	4 (36.4)	3 (27.3)	11
*M.catarrhalis* ^c^	6	3	3	8
*S. aureus* ^c^	1	1	0	2

^a^The categories of coinfection with bacteria and with viruses are not mutually exclusive

^b^Data are n (%)

^c^The percentages are not listed because the total episodes is too small

### Comparisons of clinical characteristics of the patients with single and mix infections

The demographic and clinical characteristics of the patients with single/mix infections are shown in Table 2. Their median age of children with single bacterial infections (35 months), as well as those with mixed bacterial pathogens (30 months) was significantly greater than that of children infected with single viruses (12 months) or mixed viruses (12 months) (*P* < 0.01, respectively). The duration of symptoms and usage of antibiotics preceding admission was similar among all groups of patients. The proportion of children presenting with wheezing was lowest among children with single bacterial infection, while the proportion of children presenting with fever, as well as percentage of neutrophils was lowest among children with single virus infection (all *P* < 0.01). Children with single virus infection had a higher proportion of oxygen therapy compared with single bacterial infection (11.4% vs 2.7%, *P* < 0.01), while the PICU admission rate did not differ between the two groups. The type of infection was not associated with length of hospital of stay.

**Table 2:** The Demographic and Clinical Characteristics of 593 Patients with Community-acquired Pneumonia Associated with Single/mix infections

Characteristics	Single virus	Single bacteria	Mixed viruses	Mixed bacteria/viruses	Mixed bacteria	P value
No. of patients	118	182	28	209	56	_
Age, months^a^	12^cd^	35^cef^	12^eg^	20 ^f^	30^dg^	<0.01
Gender, % male	62.8	54.0	67.9	56.9	66.1	0.10
Duration of symptoms before admission, days^b^	7.0	6.5	6	5.8	7.2	0.43
Antibiotic therapy during preceding 2 weeks, %	76.3	70.2	73.2	66.6	70.5	0.21
Fever, %	35.6^cdef^	67.5^cg^	51.8^dg^	53.7^e^	57.3^f^	<0.01
Wheeze, %	50.8^c^	32.4^cdef^	51.9^d^	47.8^e^	44.1^f^	<0.01
Dyspnea, %	9.0	7.9	7.7	8.5	8.1	0.41
WBC count, ×10^9^/L^b^	11.3	10.2	11.9	11.3	11.8	0.25
Percentage of neutrophils^b^	34.9^cde^	47.9^c^	40.1	45.2^d^	49.4^e^	<0.01
CRP, mg/L^b^	10.6	13.6	22.5	14.3	15.6	0.07
Pleural effusion, %	1.5	3.2	3.7	2.5	1.5	0.64
Need of oxygen, %	11.4^c^	2.7^cd^	11.1	9.5^d^	8.1	0.02
PICU admission, %	0^c^	0.6	7.4^c^	3.5	2.2	0.01

^a^The median value was used

^b^The mean value was used

^c^Significant differences were observed between each pair of values

^d^Significant differences were observed between each pair of values

^e^Significant differences were observed between each pair of values

^f^Significant differences were observed between each pair of values

^g^Significant differences were observed between each pair of values

We further performed multivariate logistic-regression analyses to determine the best predictors of mix infections. Multivariate logistic-regression analyses revealed that only 2 variables were associated with mix infections: age < 6 months (odds ratio: 2.1; 95% confidence interval: 1.2–3.3) and admission of PICU (odds ratio: 12.5; 95% confidence interval: 1.6–97.4). Hosmer–Lemeshow statistic was 0.51, which indicated a lack of deviation between the model and observed event rate. The AUROC for age was 0.71 (95% CI, 0.58-0.92), and for admission of PICU was 0.67 (95% CI, 0.54-0.88).

## Discussion

Eight hundred forty-six children immunocompetent children hospitalized were studied prospectively to elucidate the etiologic spectrum and occurrence of coinfections. A comprehensive investigation combining microbiologic, serologic, and molecular tests was undertaken to maximize the diagnostic yield. Overall, a pathogen was identified in 76.5% of children. Coinfection was identified in 34.6% of the patients.

The most commonly detected pathogens in our study were RSV (22.9%) and HRV (22.1%). In a recent population-based surveillance in USA, RSV was detected among 28% of children and HRV was 27% detected among <15 years old hospitalized with pneumonia similar to our results [6]. In another 6-year prospective study conducted in children <14 years, the most frequently detected virus was RSV with 41.6% of positive patients followed by RV (26.2%), which was also in line with our study [13].

*S. pneumoniae* was detected in 3% of the study population, which is similar with a recent study in USA (4%) [6], but lower than an earlier US pediatric pneumonia etiology study. The 7-valent pneumococcal conjugate vaccine was introduced to China in 2008, it has been covered in around 15% of the children in the last seven years [14]. While our data, as well as the recent study in USA [6], partly reflect the substantial reduction of pneumococcal disease due to conjugate vaccines, bacterial culture-based diagnostics have limited sensitivity and bacteremia is detected in a minority of pneumococcal pneumonias [6].

In our study, the proportion of mix infection was 34.6%, the majority (71.3%) of which were mixed viral-bacterial infections. Our study is similar to a previous study which had a mix infection rate of 35%, they also found that most of the coinfections were mixed viral-bacterial infections [11]. Michelow et al. conducted a prospective diagnostic study and found that mix infection rate was 34%, they also found that the majority of which were mixed viral-bacterial infections [10]. It has been speculated that viruses may induce pneumonia, either directly or by rendering the host more susceptible to bacterial infection. The high prevalence of mixed viral-bacterial infection found in our study, as well as the previous studies raise the important question of whether sequential or concurrent viral and bacterial infections have a synergistic impact on the evolution of disease in children [15]. However, in Jain et al. study, they found that the proportion of pathogen co-detection was 26%, the majority of which were mixed viral-viral infections [6]. The difference of coinfection rate and pattern of coinfection may be related to seasonal, geographical, and racial factors.

In our study, children with proven single/mix viral infections tended to be younger than children with single/mix bacterial infections. This is also approved in Michelow’s study [10]. In their study, they also found that the proportion of children presenting with wheezing was lowest among children with bacterial infection, while the proportion of children presenting with fever, as well as percentage of neutrophils, was lowest among children with virus infection. However, in their study, they neither compared the clinical characteristics between single and mix virus infection, nor compared between single and mix bacterial infection. Interestingly, in our study, children with mix viral infections presented with a higher proportion of fever compared with those with single viral infection, which suggested that a combination of virus infections had more degree of inflammation than single virus infection. Besides, we also found that children with mix bacterial infections presented with a higher proportion of wheeze compared with those with single bacterial infection. The higher proportion of wheeze in children with mix bacterial infections may be attributed to the protracted bacterial bronchitis (PBB). PBB is disease characterized by protracted wet cough >4 weeks and bacterial culture-positive BALF. A high proportion of wheezing episodes was reported in children with PBB [16, 17]. Our previous study found that wheezing was presented in 90% of the children with PBB [18]. Actually, in our study, 11 patients were diagnosed as PPB. Nine of the 11 patients had the mix bacterial infection.

To date, the relationship between the clinical severity and infection status with single vs. multiple respiratory pathogens remains inconclusive. Asner et al. Reported that equivalent clinical severity was observed between children with single virus infection and virus coinfection [19]. In adult, previous works also pointed that CAP patients requiring ICU admission did not point out any relationship between viral-bacterial coinfection and severity [20, 21]. However, other studies found that coinfection was associated with higher rate of intensive care unit admission and receipt of mechanical ventilation as well as longer hospital stay in children and adult [22–26]. In our study, we found that patients with mix infection had a higher rate of PICU admission, while the type of infection was not associated with length of hospital of stay. Further studies with larger populations may explore this point, with comparing the severity and prognosis of CAP patients according to the type of virus as well as the different type of virus/bacteria combinations.

In our study, 73 patients (8.6%) infected with three or four pathogens. Interestingly, compared with 220 (75.1%) patients with pathogens, children with three or four pathogens had a longer duration of hospital stay, after adjusted by age (*P* < 0.01), while oxygen therapy or the PICU admission rate did not differ between the two groups (data not shown). This interesting phenomenon may reflect the fact that patients with multiple pathogen infection has a more effective mechanism for evading the innate immune response, resulting in slower pathogenic clearance and greater lung injury.

Our study has some limitations. As previous study documented [27–29], detection of pathogens in the upper respiratory tract may not represent causation of pneumonia. Although thoracocentesis may provide more solid evidence of pathogens, invasive procedures were not commonly performed due to feasibility considerations. Second, prevalence of each pathogens varied yearly, their association with CAP may also variable. Third, the tests used for virus detection were heterogeneous, as some were detected by immune-fluorescence and some other by PCR. Fourth, our virus tests did not cover all the common viruses, such as coronaviruses, which had positive rate of around 3% in patients with acute respiratory infections in a recent Chinese study [30]. Finally, although our study investigated large populations with standardized procedures, our findings in a single center may not be representative of the entire Chinese pediatric population.

## Conclusion

In conclusion, virus accounted for the majority of identifiable infections in children hospitalized with CAP in this series. Effective anti-viral vaccines or treatments, particularly for RSV and HRV could have an impact on pediatric pneumonia. Mixed infections were identified in 34.6% of the patients with CAP. The majority of these were mixed viral-bacterial infections. The high mix infection burden in childhood CAP underscores a need for the enhancement of sensitive, inexpensive, and rapid diagnostics to accurately identify pneumonia pathogens.

## Abbreviations

ADV: Adenovirus
BAL: Bronchoalveolar
CAP: Community acquired pneumonia
DFA: Direct immunofluorescence assay
hMPV: Human metapneumovirus
HRV: Human rhinoviruses
IVA: Influenza virus A
IVB: Influenza virus B
OD: Optical density
PBB: Protracted bacterial bronchitis
PCR: Polymerase chain reaction
PICU: Pediatric intensive care unit
PIV: Parainfluenza virus
RSV: Syncytial virus infection
SD: Standard deviation
